# Bacterial-Specific Aggregation and Killing of Immunomodulatory Host Defense Peptides

**DOI:** 10.3390/ph14090839

**Published:** 2021-08-24

**Authors:** Nauman Nazeer, Juan Carlos Rodriguez-Lecompte, Marya Ahmed

**Affiliations:** 1 Department of Chemistry, University of Prince Edward Island, Charlottetown, PE C1A 4P3, Canada; nnazeer@upei.ca; 2 Department of Pathology and Microbiology, Atlantic Veterinary College, University of Prince Edward Island, Charlottetown, PE C1A 4P3, Canada; jrodriguez@upei.ca; 3 Faculty of Sustainable Design & Engineering, University of Prince Edward Island, Charlottetown, PE C1A 4P3, Canada

**Keywords:** antibacterial peptides, bacterial flocculation, nonhemolytic, immunomodulatory

## Abstract

This study involves the design and development of disulfide bridge-linked antimicrobial peptides using the host defense protein Angiogenin 4 (chAng4) as a template. The mini peptides derived from chAng4 (mCA4s) were evaluated for their antibacterial efficacies in various pathogenic bacterial strains, and the role of the oxidation state of thiols in the peptide sequence and its implication on antibacterial properties were explored. A remarkable property of these synthetic mCA4 peptides is their capability to flocculate bacteria and mediate bacterial-specific killing, in the absence of any other external stimulus. mCA4s were further evaluated for their cellular uptake, hemolytic activities, toxicities, and immunomodulatory activities in different eukaryotic cell lines. The results indicate that disulfide bridge-containing cationic amphipathic peptides show superior antibacterial efficacies, are nontoxic and nonhemolytic, and mediate bacterial flocculation and killing, in the absence of external stimuli.

## 1. Introduction

Host defense peptides (HDPs) are low-molecular-weight (2–50 kDa) fragments of proteins (made up of 12–50 amino acids) that are present in the innate immune system of a variety of organisms including plants, insects, bacteria, fungi, and viruses, as a first line of defense against microbial infections [1]. Since the discovery of the first HDPs in 1970s, more than 2000 peptides have been identified in different organisms and are being evaluated for their potential as antimicrobial agents, as well as for their immunomodulatory activities [2,3].

HDPs are generally defined as cationic and amphipathic with bacterial killing and immunomodulatory properties; however, one of the remarkable and less explored properties of HDPs is their inherent propensity to aggregate under physiological conditions [4]. Recently, the study of aggregation propensity of natural and synthetic cationic amphipathic peptides is gaining great interest in the literature to develop novel bacterial selective antimicrobial drug candidates. For example, the aggregation capability of peptides has been used as a method to increase the selectivity of peptides toward bacterial membranes, thereby causing selective toxicity in prokaryotic cells [5]. However, truncated derivatives of larger HDPs have been reported to lose antibacterial activity at smaller sizes and lower cationic charges [6,7,8,9,10,11,12,13,14,15]. Most of these bacterial-selective peptides engineered by fusing multiple peptide sequences are, therefore, much larger in size (>15 amino acids) and are not a cost-effective option as antimicrobial agents. For example, precisely designed sequences of human defensin-6 are developed by fusing a ligand-binding sequence with β-sheet assembled peptides. The engineered peptides self-assemble in the presence of *S. aureus* into fibrous networks that entrap bacteria and block invasion and infections in living tissues [16].

To the best of our knowledge, enzyme-sensitive tryptophan (Trp)-rich peptides are the only example of short synthetic peptides (<10 amino acids) that can interact with bacteria via Trp-rich moieties and can aggregate them into flocs in the presence of enzymes. The enzyme-responsive dephosphorylation of peptides in the presence of bacteria leads to the formation of self-assembled structures that increase the net cationic charge on the surface of bacterial membranes and contribute to the bacterial killing [17]. However, unlike HDPs, the immunomodulatory efficacies of these short peptides are unknown.

**Scheme 1:**
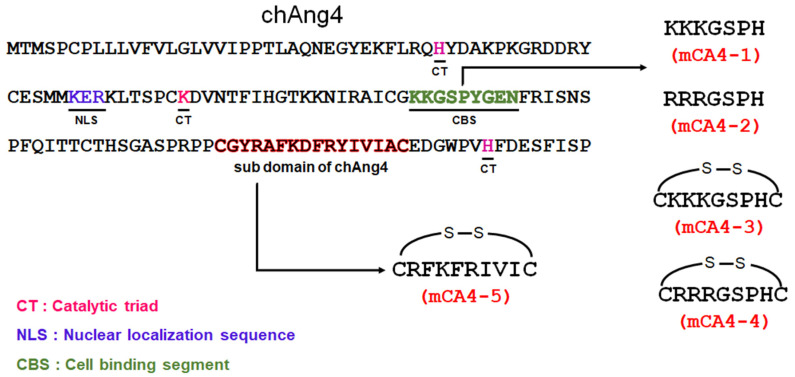
Schematic depicting the design of mCA4s from chicken host defense protein.

Given the significant and proven potential of peptides as emerging antimicrobial and immunomodulatory compounds and the emerging interest in flocculation and selective bacterial killing efficacies of antimicrobial peptides, we report the design and synthesis of short cationic and amphipathic peptides with potential immunomodulatory, bacterial flocculation, and antibacterial activities. The novel peptides were derived from chicken Angiogenin 4 (chAng4) and are termed as mini peptides derived from ChAng4 (mCA4s). chAng4 is a 144 amino acid long HDP that was first reported by Rodriguez-Lecompte in the intestines of chickens [18]. Different mCA4s are generated by combining the catalytic triad component with the truncated cell-binding sequence of a protein and by the truncation and amino-acid substitution of the subdomain of chAng4 (Scheme 1). The short cationic and amphipathic mCA4s (<10 amino acids) produced were evaluated for their antibacterial activities in a broad range of Gram-positive and -negative bacteria, and the antibacterial efficacies of cysteine-free peptides with the cysteine-containing peptides were compared. The oxidation state of thiols in the peptide structure was also evaluated, and the effect of the oxidation state of thiols on the antibacterial efficacies of peptides was studied. Our results indicate that disulfide bridge-containing cationic amphipathic peptides exhibited superior antibacterial efficacies in a range of Gram-positive and Gram-negative bacteria and have the potential to contain bacterial infections by flocculation in the presence of bacteria. The disulfide bridge-containing cationic amphipathic peptides were also immunomodulatory and suppressed the production of inflammatory cytokines produced by bacterial toxin-challenged macrophages. The short synthetic peptides were also taken up well and are nonhemolytic and nontoxic toward eukaryotic cells (including epithelial, fibroblast, and red blood cells), during in vitro studies.

## 2. Results and Discussion

### 2.1. Peptide Synthesis and Characterization

The synthetic short peptide sequences (<10 amino acids) of chAng4 were prepared by SPPS via Fmoc protection/deprotection chemistry (Table 1). mCA4-1 and its analog mCA4-2 prepared by substitution of lysine into arginine are hydrophilic peptides that were derived by truncation and residue substitution of cell-binding segments of the chAng4 (Scheme 1) [19]. The introduction of thiol groups at the two ends of these short peptides yielded mCA4-3 and mCA4-4, and this was used as a strategy to facilitate the cellular uptake and to improve the antimicrobial activity of highly hydrophilic cationic peptides by incorporating disulfide cyclization [20,21]. mCA4-5, a cationic amphipathic AMP, was also designed from truncated segments of chAng4 and contained alternately arranged hydrophilic (R and K) and hydrophobic amino acids (F, I, and V). The hydrophilic cationic peptides were purified by precipitation in organic solvent and exhibited reduced purity (Supplementary Figure S1). The cationic amphipathic peptide synthesized was purified by RP-HPLC. The mass of the peptides was analyzed by electrospray ionization mass spectrometry (ESI-MS), and the predicted molecular weights of mCA4-1, mCA4-2, mCA4-3, mCA4-4, and mCA4-5 (779.5, 863.5, 985.49, 1069.51, and 1282.72 g/mol, respectively) were in agreement with the experimentally obtained molecular weights by ESI-MS, as shown in Table 1 (Figure S1). The summary of synthesized peptides is listed in Table 1. The synthesized AMPs possessed a net positive charge of +3 and were mainly hydrophilic in character. The cationic amphipathic peptide mCA4-5 contained 50% hydrophobic character and was eluted at 23% *v*/*v* acetonitrile concentration during reverse-phase HPLC (Figure S2). The designed peptides meet the basic reported classification of antimicrobial peptides (AMPs), i.e., an overall net positive charge (minimum of +2), presence of thiols in the peptide structure, and an amphipathic structure that is present for at least one peptide sequence of mCA4 [22]

### 2.2. Bacterial Toxicity and Uptake

The purified peptides were tested for their antibacterial efficacies in Gram-negative and Gram-positive bacteria. Our preliminary data indicated that mCA4-1, -2, and -3 were inactive against all tested bacterial strains, indicating that an overall net positive charge of +3 and the presence of thiols in a hydrophilic peptide structure are not sufficient to yield antimicrobial activities. mCA4-4 showed species-specific antibacterial activity against *E. coli, S. enterica*, and *S. aureus* at relatively high concentrations of peptides (25–70 µM), indicating that substitution of lysine with arginine in the peptide sequence indeed improved the antibacterial efficacies of peptides. However, mCA4-4 was found to be completely inactive against *L. monocytogenes* at all studied concentrations (up to 100 µM). mCA4-5, the cationic amphipathic peptide, showed potent antibacterial activity against all studied bacteria, including pathogenic strains of *S. aureus*, *L. monocytogenes*, and *S. enterica* (Table 2), and antibacterial activities of this peptide were similar or superior to ampicillin, which was used as a positive control. mCA4-5 was especially effective against *L. monocytogenes* (IC_50_ = 5.2 µM; MIC = 10 µM) and showed 2–3-fold higher antibacterial efficacies than ampicillin (IC_50_ = 11.2 µM; MIC = 32 µM). The superior antibacterial activities of mCA4-5 are attributed to its cationic amphipathic character and are in agreement with the strong bacterial toxicities of other cationic amphipathic peptides reported in the literature in a low micromolar range [6,7,8,9,10,11,12,13,14,15].

To understand the difference in antibacterial activity of two peptides, the uptake of fluorescently labeled cationic (mCA4-4) and cationic amphipathic (mCA4-5) peptides in Gram-negative and Gram-positive bacterial models *E. coli* and *B. subtilis*, respectively, was first studied at a sublethal concentration of peptide. The bacteria were treated with TAMRA-labeled peptides at 5 µM concentration for 5 h, and cellular uptake of the peptides was recorded as a function of time (Supplementary Figure S3). TAMRA-labeled cationic and cationic amphipathic peptides were taken up well in both *E. coli* and *B. subtilis*, and the maximum uptake of peptides in bacteria was achieved over a 5 h time period. The cationic amphipathic peptide mCA4-5 specifically showed rapid uptake in bacteria, possibly due to the superior interactions of the peptide with lipophilic bacterial membranes [6,7,8,9,10,11,12,13,14,15,22]. The overall results suggest that the difference in IC_50_ values of the two peptides was not directly related to their difference in bacterial uptake. These results are consistent with the recent literature indicting that a high uptake of cationic peptides in bacteria does not always contribute toward bacterial toxicity, and this has been used as a strategy to deliver antibiotics in bacterial cells [10,23,24].

### 2.3. Membrane Permeabilization

We next studied bacterial outer membrane permeabilization capability in the presence of peptides and membrane-impermeable fluorescent dye 5(6)-carboxyfluorescein (CF). *E. coli* and *B. subtilis* were incubated in the presence of different concentrations of peptides, and the uptake of membrane-impermeable fluorescent dye in bacterial cells, as a function of time and peptide concentration, was evaluated (Figure S4). As expected, mCA4-5-treated bacteria showed a significant uptake of CF as a function of concentration of peptide, and the uptake of dye plateaued at 50 µM concentration, possibly due to the significant bacterial death at an elevated concentration of peptides (Figure S4A,C). The incubation of bacteria at a sublethal concentration of peptide (IC_50_ value) in the presence of CF dye caused a rapid increase in the fluorescence of bacterial cells over time, indicating that membrane destabilization is likely the mechanism of action of mCA4-5 (Figure S4B,D). mCA4-4, however, showed poor membrane permeabilization efficacies in *E coli* and only slight membrane permeabilization effects were observed in Gram-positive bacteria at very high concentrations (above 80 µM) of peptide. The membrane permeabilization capability of cationic peptide at a very high concentration is consistent with the high IC_50_ values of mCA4-4 in bacteria, indicating that a high concentration of this peptide is required to compromise the membrane permeability of bacterial cells.

### 2.4. Role of Disulfide Bridges in Antibacterial Activity

mCA4-4 and -5 are cationic and amphipathic peptides that contain cysteines at the terminal ends of each peptide sequence. The presence of cysteine in the peptide sequence is well documented to improve cellular uptake and to enhance antibacterial efficacies [20,21]. In our study, the addition of cysteine indeed improved the antibacterial bacterial efficacies of mCA4-4 as compared to its cysteine-free version. To understand the role of oxidation state of thiols in peptide structures, Ellman’s assay was first performed to analyze the presence of free (reduced) thiol groups in the peptide structure (Figure S5). Thus, 40 µM peptide samples were treated with Ellman’s reagent, and the absorbance of samples was recorded at 412 nm. _L_-Cysteine hydrochloride monohydrate was used as a control to create a calibration curve as a function of the concentration of thiols. Interestingly, mCA4-4 and -5 did not yield any absorbance at 412 nm, indicating that cysteines present in peptide structures mainly exist in the oxidized form, and that cyclic mCA4-4 and -5 are potent antibacterial agents against a variety of different bacteria. To further explore the role of disulfide bridges in the antibacterial activities of peptides, IC_50_ concentrations of mCA4-4 and -5 were incubated with *E. coli* in the presence of 2 mM glutathione (GSH), and the antibacterial activities of reduced peptides were studied (Figure S6). Interestingly, the treatment of mCA4-4 with GSH significantly reduced the antibacterial activities of the peptide. GSH-treated mCA4-4 maintained up to 75% bacterial viability, while the viability of *E. coli* was only 15% in the absence of GSH (oxidized form of peptide), suggesting that the oxidized structure of cationic hydrophilic peptide is mainly responsible for its superior antibacterial activity. In contrast, treatment of cationic amphipathic peptide (mCA4-5) with GSH had only a marginal effect on the antibacterial activity. The percentage bacterial viability changed from 9% to 27% upon GSH treatment of mCA4-5, indicating that disulfide bridges only tend to slightly improve the activity of cationic amphipathic peptide. 

The presence of thiols in natural AMPs and organometallic antimicrobials is well documented to broaden the antimicrobial spectrum of these peptides; however, the incorporation of thiol in preformed peptide structures is costly. The presence of free thiols in a peptide sequence is also known to cause hemolytic activity and nonspecific toxicity against eukaryotic cells [25]. The toxicity and hemolytic activity of our cyclic peptides was further investigated to ensure the safety of peptides toward eukaryotic cells.

### 2.5. Cellular Uptake, Cytotoxicity, and Hemolytic Activities

**Figure 1:**
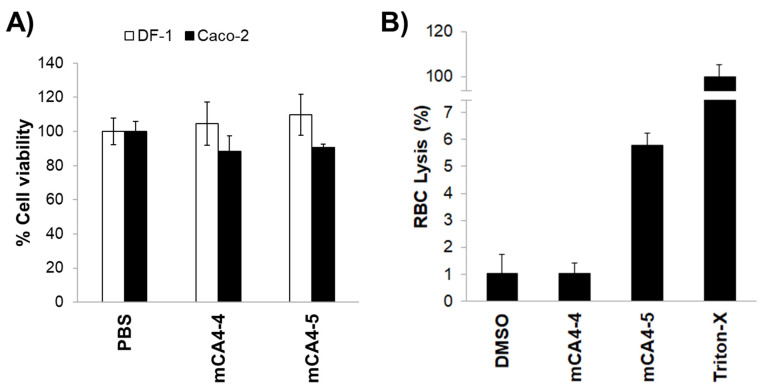
(**A**) Cell viability of DF-1 fibroblasts and Caco-2 cells upon incubation with 100 µM peptides for 24 h; (**B**) percentage hemolysis in red blood cells upon treatment with 100 µM peptides for 4 h. Error bars indicate the standard deviation of triplicates. Percent hemolysis in red blood cells upon treatment with 100 µM peptides for 4 hours. DMSO is used as a negative control and Triton-X100 is used as a positive control and indicate 100% red blood cell lysis.

The toxicity of disulfide bridge-linked cationic and cationic amphipathic peptides was evaluated in intestinal epithelial cells and in fibroblasts. DF-1 fibroblasts and Caco-2 cells were treated with 100 µM concentrations of mCA4-4 and -5 for 24 h, and the toxicity of peptides toward eukaryotic cells was measured by MTS assay (Figure 1). As shown in Figure 1A, treatment of fibroblasts and intestinal epithelial cells at 100 µM peptide concentration did not compromise the viability of eukaryotic cells. The hemolytic activity of peptides was then tested by treating the red blood cells with 100 µM mCA4-4 and -5. The peptides showed negligible hemolytic activity (<6%) toward red blood cells, even after 4 h of incubation, suggesting that the oxidized state of thiols in a peptide structure does not impart nonspecific toxicity toward eukaryotic cells. The cyclic peptide mCA4-5 is an example of a potent antibacterial agent with a broad range of antibacterial activities but is nontoxic and is hemocompatible toward eukaryotic cells, even at concentrations severalfold (>6-fold) higher than the IC_50_ of the peptide.

**Figure 2:**
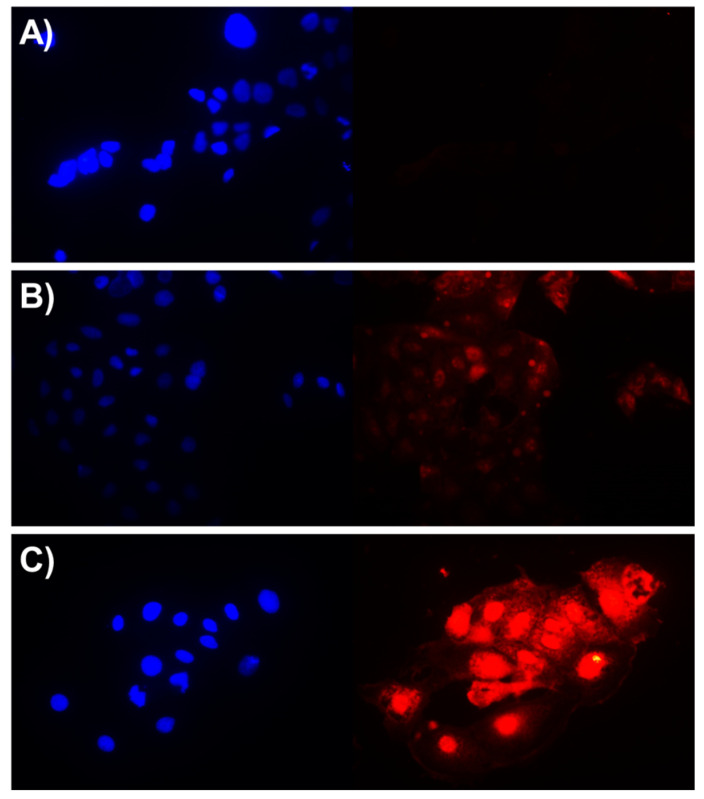
Fluorescence microscopy images of Caco-2 cells treated with IC_50_ values of TAMRA-labeled peptides for 5 h: (**A**) untreated control; (**B**) TAMRA-labeled mCA4-4; (**C**) TAMRA-labeled mCA4-5.

The cellular uptake of peptides was then studied in eukaryotic cells. TAMRA-labeled peptides were incubated with Caco-2 cells for 5 h, and the uptake of peptides was studied by flow cytometry and fluorescent microscopy (Figure 2, Figure S7). In comparison to mCA4-4 that showed only slight uptake in Caco-2 cells (19% fluorescent cells), mCA4-5 was highly taken up in eukaryotic cells (33% fluorescent cells), indicating that the cationic amphipathic peptide mediates superior interactions with biological membranes [6,7,8,9,10,11,12,13,14,15].

The uptake of fluorescent peptides in Caco-2 cells was visualized by fluorescence microscopy (Figure 2). As shown in Figure 2, mCA4-5 is well taken up, and the peptide tends to accumulate mainly in the nucleus of cells, in contrast to the cationic mCA4-4, which shows weak fluorescence in Caco-2 cells. The microscopy images obtained are in agreement with flow cytometry data and suggest the superior membrane permeabilization efficacies of mCA4-5 in Caco-2 cells; however, the interactions of peptides with biological membranes do not translate into cell death.

### 2.6. Intracellular Bacterial Treatment 

**Figure 3:**
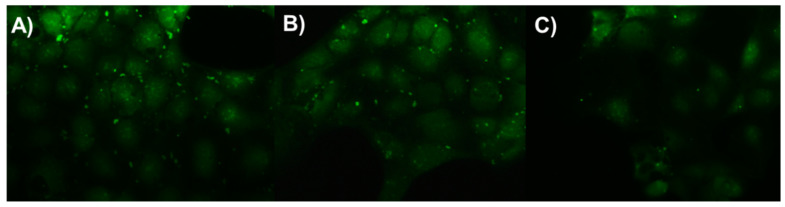
Bacterial neutralization efficacies of peptides: (**A**) untreated; (**B**) 5 µM mCA4-4; (**C**) 5 µM mCA4-5. The fluorescence images were taken upon incubation of GFP-expressing bacteria with peptide samples for 5 h.

Given the superior antimicrobial efficacies and the nontoxic and hemocompatible behavior toward eukaryotic cells, we then evaluated the antibacterial activities of these peptides to prevent bacterial infections in eukaryotic cells. Caco-2 cells were infected with 400,000 CFU/mL of GFP-expressing *E. coli* and were treated with 5 µM of peptides for 5 h. The sub-IC_50_ concentration of mCA4-5 was used for the treatment of bacterial infection, as the reduced number of bacteria CFU/mL was required to obtain optimal infection in eukaryotic cells [26]. As shown in Figure 3, in the absence of peptides, fluorescent *E. coli* rapidly invaded Caco-2 cells. mCA4-4 was unable to reduce the bacterial burden at low concentrations; however, cotreatment of mCA4-5 with bacteria actively contained and reduced bacterial infections in mammalian cells. This bacterial-neutralizing capability of AMPS is typically attributed to the preferential binding of cationic amphipathic peptides to bacterial membranes than eukaryotic cells and is documented to cause rapid bacterial killing before the internalization of pathogens in eukaryotic cells [26].

### 2.7. Bacterial Flocculation Capability

**Figure 4:**
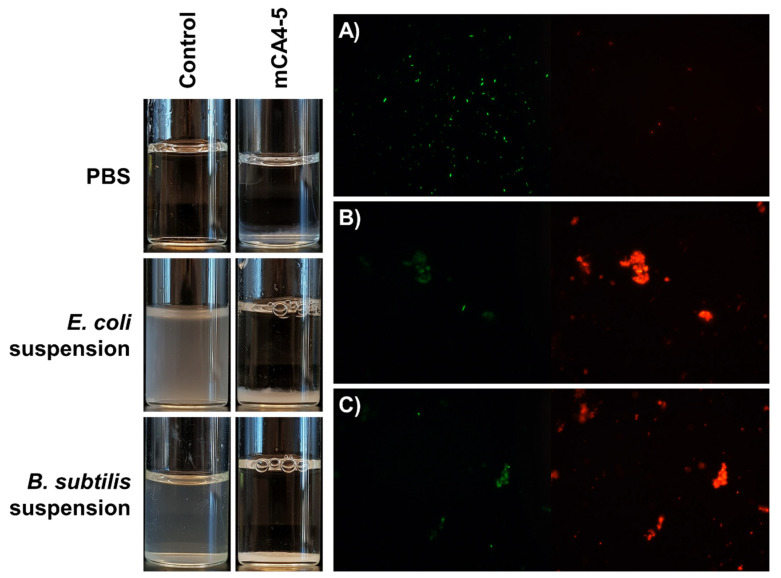
Optical images of PBS solution, *E. coli* suspension, and *B. subtilis* suspension in the presence and absence of peptide (**left**)**.** Fluorescence microscopy images (**right**) of (**A**) PBS-treated *E. coli*, (**B**) peptide-treated *E. coli*, and (**C**) peptide-treated *B. subtilis*.

Another remarkable property of mCA4-5 peptide, which was not observed in the other mCA4s, was its ability to flocculate bacteria in the form of clumps (Figure 4). As shown in optical images, *E. coli* and *B. subtilis* are well dispersed in phosphate-buffered saline at room temperature and in the absence of peptide. The addition of micromolar concentrations of mCA4-5 causes significant agglutination of bacteria in the form of clumps that settle down slowly over a period of 3 h. The viability of bacteria in flocs was then studied, and clumps of dead bacteria (red clumps) were visible under a fluorescence microscope. The aggregates of dead bacteria indicate that the cationic amphipathic peptide can entrap bacteria in flocs, thereby increasing the concentration of peptides on bacterial membranes and causing cell death. This bacterial aggregation capability has also been reported in some natural AMPs that specifically contain bacterial-targeting domains. These peptides tend to promote the accumulation of peptides on the bacterial membranes, thus increasing local peptide concentration against targeted microbes [27]. Recently, tryptophan-based synthetic cationic self-assembled peptides have shown similar behavior in terms of bacterial flocculation and cell death. The interactions of Trp with bacterial cells in the presence of enzyme-responsive peptides yield self-assembled structures that can agglomerate and cause bacterial death [17]. mCA4-5 is a unique short cationic amphipathic peptide that does not specifically contain bacterial ligand-binding domains and, unlike natural HDPs, does not agglomerate itself in saline solution, but specifically agglomerates in the presence of bacteria and in the absence of any other stimulus.

### 2.8. Neutralization of Bacterial Toxins and Cytokine Release Studies

mCA4-4 and -5 are fragments of Angiogenin 4, a protein that is well documented for its immunomodulatory role to combat bacterial infections [28]. The synthetic mini peptides (mCA4-4 and mCA4-5) were further tested for their ability to neutralize bacterial toxins (LPS and LTA) in vitro and for the induction of cytokines in macrophages. Caco-2 cells were treated with LPS (*E. coli*-derived endotoxin) and LTA (*S. aureus*-derived exotoxin) in the presence and absence of synthetic peptides (100 µM), and cell viability was tested by MTS assay. Although mCA4-4 and -5 showed superior antimicrobial activity and were nontoxic toward eukaryotic cells, the peptides showed limited capability to neutralize bacterial toxin (Supplementary Figure S8). mCA4-5 specifically showed only marginal LPS neutralization capabilities, and LPS-treated Caco-2 cell viability was improved from 30% to 46% in the presence of mCA4-4. Similar results were observed upon LTA treatment of Caco-2 cells, and cell viability was improved by only 15% in the presence of peptides, suggesting that the peptide structures were not optimal for binding and neutralization of bacterial toxins during in vitro studies.

**Figure 5:**
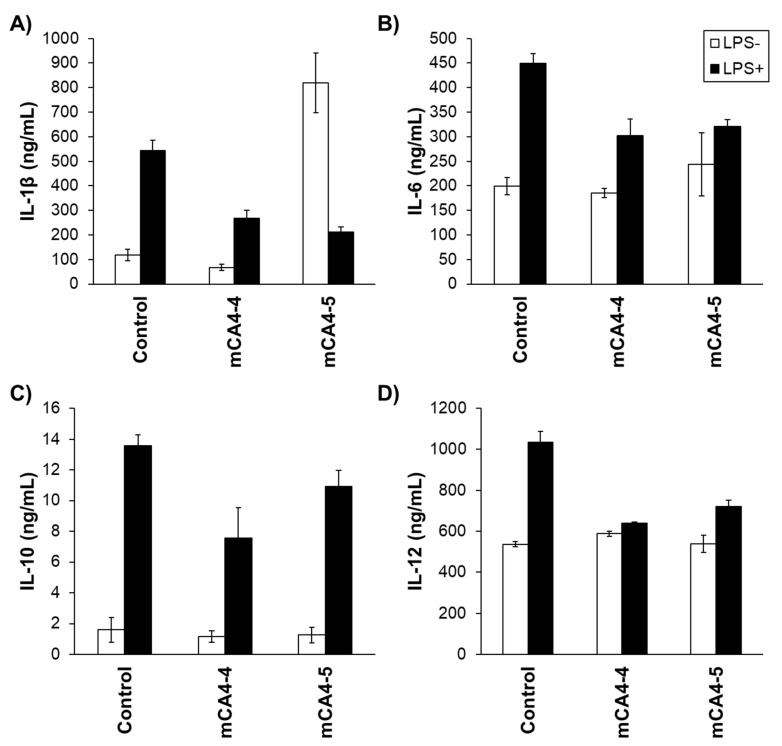
Release of cytokines (**A**) IL-1β, (**B**) IL-6, (**C**) IL-10, and (**D**) IL-12 from HD11 macrophages treated with LPS and 100 µM peptides. LPS+ indicates macrophages stimulated by 1 µg/mL LPS, and LPS− indicates unstimulated macrophages. Error bars indicate the standard deviation of triplicates. White bar indicates the treatment of macrophages with peptides in the absence of LPS and black bar indicate the treatment of macrophages in the presence of LPS.

The role of peptides in cytokine production was then evaluated. The macrophages were treated with the peptides in the presence and absence of bacterial toxins, and the production of inflammatory cytokines was evaluated (IL-1β, IL-6, IL-10, and IL-12) by ELISA assay (Figure 5 and Supplementary Figure S9). As shown in Figure 5, in the absence of peptides, significant overexpression of inflammatory cytokines was observed upon treatment with LPS. The treatment of macrophages with mCA4-4, in the absence of LPS, did not cause any significant overexpression of all studied cytokines, suggesting the inert and nonmodulatory nature of the cyclic cationic peptides. The treatment of LPS-free macrophages with mCA4-5, however, resulted in strong overexpression of IL-1β, indicating the proinflammatory nature of this peptide. However, in the presence of LPS, both mCA4-4 and -5 could efficiently inhibit the LPS-induced production of proinflammatory cytokines in HD11 macrophages. These results are consistent with a recent study, where natural HDP-derived bacterial flocculating peptides exhibited the suppression of IL-1β from LPS-stimulated macrophages. Although the suppression of LPS-induced inflammation has been observed in various natural [29], synthetic [30,31], and chimeric AMPs [32,33], the notable feature of mCA4-5 is its reduced length, reduced charge, and bacterial-flocculating efficacy, when compared with many other immunomodulatory AMPs.

## 3. Materials and Methods

### 3.1. Materials

Rink amide AM resin Fmoc-protected amino acids and HBTU were purchased from Matrix Innovation Inc. (Québec, QC, Canada), *N,N*-Diisopropylethylamine (DIPEA), acetonitrile, trifluoroacetic acid (TFA), 5(6)-carboxyfluorescein, 0.25% trypsin-EDTA and other reagents were obtained from Sigma Aldrich (Oakville, ON, Canada) unless indicated otherwise. *Escherichia coli* ATCC 25922, *Staphylococcus aureus* ATCC 25923, *Salmonella enterica* ATCC 13076, *Listeria monocytogenes* ATCC 19115, DF-11, HD-11, and CaCo-2 were purchased from Cedarlane (Burlington, ON, Canada). 5(6)-Carboxytetramethylrhodamine (TAMRA) was obtained from Fisher Scientific (Ottawa, ON, Canada). Dulbecco’s modified Eagle’s medium (DMEM), Eagle’s minimal essential medium (EMEM), fetal bovine serum (FBS), and 0.25% trypsin-EDTA were purchased from VWR Life Sciences (Mississauga, ON, Canada). Chicken serum (CS) was purchased from Gibco (Dublin, Ireland). CellTiter 96^®^ AQueous Cell Proliferation Assay was purchased from Promega (Madison, WI, USA).

### 3.2. Peptide Synthesis and Characterization

Peptides were synthesized by solid-phase peptide synthesis (SPPS) using a Focus XC Peptide Synthesizer (AAPPTec) via Fmoc protection/deprotection chemistry, using rink amide resins, HBTU as an initiator, and DIPEA as a catalyst. Peptides were purified using a reverse-phase high-performance liquid chromatography (HPLC) system, Agilent Technologies (Santa Clara, CA, USA), with water and acetonitrile containing 0.1% TFA as the mobile phases. 

Liquid chromatography/high-resolution mass spectrometry (LC-HRMS) was used to determine the molecular weights of the synthesized peptides. Mass spectrometry data were provided by the AIMS Mass Spectrometry Laboratory at the University of Toronto.

### 3.3. Antibacterial Assay

*E. coli*, *S. aureus*, and *S. enterica* were cultured in nutrient broth at 37 °C. *Listeria* was cultured in brain heart infusion broth at 37 °C. Bacteria at early log phase (OD_600_ ≈ 0.1) were incubated with the peptides at different concentrations (100–3.12 µM) for 4–6 h. After the incubation period, the bacterial culture medium was diluted at different ratios (1 in 2000–2,000,000) in sterile PBS and was spread on blood agar (BA) plates. BA plates were incubated at 37 °C for 24 h, and the number of colonies formed was counted to determine cell viability.

### 3.4. Antibacterial Activity of Glutathione-Treated Peptides 

*E. coli* was also grown in NB medium containing 2 mM glutathione (GSH). Bacteria at early log phase (OD_600_ ≈ 0.1) were then treated with mCA4-4 and mCA4-5 at concentrations of 50 and 10 µM, respectively. After an incubation period of 4 h, the cultures were plated to determine the number of colonies and cell viability.

### 3.5. Membrane Permeability Assay

*E. coli* NEB 5α and *Bacillus subtilis* ATCC 6051 were cultured overnight in nutrient broth at 37 °C. Bacterial cells were centrifuged and resuspended in PBS to an OD_600_ of 0.9 to 1. Cells in suspension were treated with 5(6)-carboxyfluorescein (CF) at a concentration of 0.5 µM and were then incubated with different concentrations of peptides (6.25–100 µM). Aliquots of the cell suspension at different time periods (up to 4 h) were centrifuged and washed twice with PBS. Membrane permeation of 5(6)-carboxyfluorescein (CF) was determined in triplicate by measuring fluorescence of the resuspended pellet at excitation and emission wavelengths of 492 and 514 nm, respectively.

### 3.6. Flocculation Assay

*E. coli* NEB 5α and *Bacillus subtilis* ATCC 6051 were grown to stationary phase in NB medium. Bacteria were centrifuged down and resuspended in PBS (pH = 7.4) at OD_600_ ≈ 0.8–1. Bacteria were then treated with 16 µM mCA4-5. Optical images of bacterial suspensions were taken after 4 h. 

### 3.7. Determination of Free Thiol Groups in Peptide Structure

The presence of free thiol groups in peptide samples was determined by Ellman’s assay. Peptide samples (at 40 µM concentration) were incubated with 5,5-dithio-*bis*-(2-nitrobenzoic acid) (DTNB) for 15 min in 0.1 M sodium phosphate buffer (pH = 8) containing 1 mM EDTA, and the absorbance of samples was measured at 412 nm. A calibration curve for the assay was constructed using cysteine hydrochloride monohydrate (CHM) as the standard.

### 3.8. Bacterial Live/Dead Assay

Peptide-treated bacteria were stained using the commercial bacterial viability kit LIVE/DEAD *Bac*Light from ThermoFisher Scientific (Ottawa, ON, Canada). This kit is composed of two nucleic acid-binding fluorochromes, namely, Syto, a green fluorescent dye which penetrates both viable and nonviable cells, and propidium iodide (PI), a red fluorescent dye which penetrates nonviable cells only and quenches the fluorescence of Syto 9. Therefore, bacteria with intact cell membranes fluoresce green, while those with damaged membranes appear red, when viewed under a fluorescent microscope.

### 3.9. Cytotoxicity Assay

DF-1 chicken embryonic fibroblasts were cultured in DMEM containing 8% FBS and 2% CS. Caco-2 cells were cultured in EMEM containing 20% FBS. Cells in growth phase (~70% confluency) were incubated with a 100 µM concentration of each peptide for 24 h. Cell viability was determined in triplicate by MTS assay using the CellTiter 96^®^ AQueous Cell Proliferation Assay, as per the manufacturer’s protocol.

The cell viability of Caco-2 cells treated with a 100 µM concentration of peptides in the presence of 100 µg/mL lipopolysaccharide (LPS) derived from *E. coli* and lipoteichoic acid (LTA) derived from *S. aureus* (purchased from Sigma-Aldrich (Oakville, ON, Canada) was also determined in triplicate using the MTS assay, as discussed above.

### 3.10. Cellular Uptake Studies

TAMRA-conjugated peptides were used for cellular uptake studies. Liquid cultures of *E. coli* and *Bacillus subtilis* at log phase (OD_600_ ≈ 0.3–0.4) were incubated with TAMRA-labeled peptides (5 µM), and the percentage uptake of peptides was determined in triplicate for 5 h by measuring the fluorescence of free peptide in the supernatant of bacteria at emission and excitation wavelengths of 579 and 546 nm.

(1) $$\%\;\mathrm{Uptake}=100-\frac{\mathrm{Fluorescence}\;\mathrm{of}\;\mathrm{supernatant}}{\mathrm{Initial}\;\mathrm{fluorescence}\;\mathrm{of}\;\mathrm{sample}}\times 100\%$$

Caco-2 human colorectal adenocarcinoma cells were cultured to growth phase (~70% confluency) in EMEM containing 20% FBS and were treated with 5 µM of TAMRA-labeled peptides for 5 h. The uptake of fluorescently labeled peptide in Caco-2 human colorectal adenocarcinoma cells was measured by flow cytometry using BD FACS Calibur (BD Biosciences (Franklin Lakes, NJ, USA)) and by fluorescence microscopy.

### 3.11. Bacterial Infection of Caco-2 Cells

Caco-2 cells grown in six-well plates were also infected with *E. coli* (ATCC 25922GFP) at a CFU of 400,000 cells/mL. Cells were then incubated with 5 µM of TAMRA-labeled peptides for 5 h and were observed under fluorescence microscope for the presence of fluorescent bacteria.

### 3.12. Cytokine Release Studies

HD11 cells were cultured in DMEM containing 8% FBS and 2% CS. Cytokine release from HD11 chicken macrophage cells was studied by treating cells in growth phase (~70% confluency) with a 100 µM concentration of peptides in serum-free DMEM medium. Cells were treated with and without 1 µg/mL LPS. After 24 h of incubation, cell culture supernatant was collected, and the production of chicken IL-1β, IL-6, IL-10, and IL-12 (purchased from Kingfisher Biotech (St. Paul, MN, USA)) was quantified in triplicate by direct ELISA assay.

### 3.13. Hemolysis Assay

A chicken red blood cell suspension was purchased from Cedarlane (Burlington, CA). RBCs were incubated with a 100 µM concentration of each peptide at 37 °C for 4 h. Triton-X was used as a positive control to achieve total hemolysis of RBCs. Percentage hemolysis was determined in triplicate by centrifuging down the RBC suspension and by measuring the absorbance of the supernatant at 540 nm.

(2) $$\%\;\mathrm{Hemolysis}=\frac{\mathrm{Absorbance}\;\mathrm{of}\;\mathrm{sample}\;@540\mathrm{nm}\;}{\mathrm{Absorbance}\;\mathrm{of}\;\mathrm{positive}\;\mathrm{control}\;@540\mathrm{nm}}\times 100\%.$$

## 4. Conclusions

In this study, we designed and synthesized mini antimicrobial peptides from chicken Angiogenin 4 and evaluated their antimicrobial, immunomodulatory, and bacterial flocculation efficacies. Among the five peptides synthesized, two cyclic peptides were shown to be nontoxic towards host cells and had low hemolytic activity. mCA4s also showed promising antibacterial activity against various species of both Gram-positive and Gram-negative bacteria and exhibited anti-inflammatory properties by suppressing proinflammatory cytokine production in LPS-challenged macrophages. The cationic amphipathic peptide mCA4-5 specifically displayed superior antimicrobial efficacies at sub-micromolar concentrations and exhibited bacterial agglomeration in vitro. The agglomeration of mCA4-5 in the presence of both Gram-positive and Gram-negative bacteria is expected to increase the net cationic charge on the bacterial surfaces, thereby increasing bacterial membrane permeation and causing bacterial death. Furthermore, treatment of mCA4-5 with GSH showed only a marginal effect on antibacterial activities, suggesting the key role of the cationic amphipathic nature of the peptide in antibacterial efficacy. Future studies will focus on elucidating the secondary structure of the mCA4-5 peptide and developing physiologically stable peptide-based nanoparticles with potential bacterial-specific killing properties for systemic applications.

## Figures and Tables

**Table 1:** Synthesized peptides and their key parameters.

Sample	Peptide Sequences	Theoretical MW (g/mol)	Experimental MW (g/mol)	Net Charge	% ACN Elution
mCA4-1	KKKGSPH	779.5	779.5	+3	-
mCA4-2	RRRGSPH	863.5	863.5	+3	-
mCA4-3	CKKKGSPHC	985.5	985.5	+3	-
mCA4-4	CRRRGSPHC	1069.5	1069.5	+3	-
mCA4-5	CRFKFRIVIC	1282.7	1281.7	+3	23%

**Table 2:** IC_50_ and MIC concentrations of Ang4-derived peptides.

Sample Name	Peptide Sequence	IC_50_ (µM)				MIC (µM)			
		*E. coli*	*S. aureus*	*S. enterica*	*L. monocytogenes*	*E. coli*	*S. aureus*	*S. enterica*	*L. monocytogenes*
Ampicillin	–	15.2	3.6	7	11.2	26.9	6.7	13.5	32.3
mCA4-4	CRRRGSPHC	68.8	52	28.2	>100	>100	100	50	>100
mCA4-5	CRFKFRIVIC	15.1	4	10.2	5.2	40	15	30	10

## Data Availability

Data is contained within the article. Mass spectrometry data on peptides, Ellman’s assay results, membrane permeability data, flow cytometry analysis, toxin neutralization assay, and GSH treatment assay of peptides are available in Supplementary Materials. The data presented in this study are available on request from the corresponding author.

## Supplementary Materials

The following are available at https://www.mdpi.com/article/10.3390/ph14090839/s1, Figure S1: Mass spectrometry results for (A) mCA4-3; (B) mCA4-4; (C) mCA4-5; (D) mCA4-2; and (E) mCA4-1 peptides, Figure S2: RP-HPLC chromatogram for mCA4-5 peptide, Figure S3: Uptake of selected AMPs in (A) *E. coli*; and (B) *B. subtilis*, Figure S4: Membrane permeabilization efficacies AMPs in (A,C) *E. coli*; and (B,D) *B. subtilis*. (A,B) were done after a fixed time interval of 30 min. (C,D) were done using a fixed peptide concentration of 25 µM, Figure S5: Calibration of L-cysteine hydrochloride monohydrate by Elman’s assay, Figure S6: Effect of glutathione treatment on antimicrobial activity of peptides in *E. coli*. Peptide concentrations for mCA4-4 and mCA4-5 were 50 and 10 µM, respectively, Figure S7: Flow cytometry data indicating cellular uptake of peptides in Caco-2 cells, Figure S8: Toxin neutralization capability of peptides, Figure S9: ELISA standard curves of (A) IL-1β; (B) IL-6; (C) IL-10; and (D) IL-12.
