# Correction: Nazeer et al. Bacterial-Specific Aggregation and Killing of Immunomodulatory Host Defense Peptides. *Pharmaceuticals* 2021, *14*, 839

**DOI:** 10.3390/ph17101359

**Published:** 2024-10-11

**Authors:** Nauman Nazeer, Juan Carlos Rodriguez-Lecompte, Marya Ahmed

**Affiliations:** 1 Department of Chemistry, University of Prince Edward Island, Charlottetown, PE C1A 4P3, Canada; nnazeer@upei.ca; 2 Department of Pathology and Microbiology, Atlantic Veterinary College, University of Prince Edward Island, Charlottetown, PE C1A 4P3, Canada; jrodriguez@upei.ca; 3 Faculty of Sustainable Design & Engineering, University of Prince Edward Island, Charlottetown, PE C1A 4P3, Canada

## Error in Table

In the original publication [1], there was a mistake in Table 1 as published.

The correct sequence order from the N to C terminal is as follows and should be reflected in Table 1.

NH_2_-HPSGKKK-CONH_2_;

NH_2_-HPSGRRR-CONH_2_;

NH_2_-CHPSGKKKC-CONH_2_;

NH_2_-CHPSGRRRC-CONH_2_;

NH_2_-CIVIRFKFRC-CONH_2_.

The corrected Table 1 appears below.

**Table 1:** Synthesized peptides and their key parameters.

Sample	Peptide Sequences	Theoretical MW (g/mol)	Experimental MW (g/mol)	Net Charge	% ACN Elution
mCA4-1	HPSGKKK	779.5	779.5	+3	-
mCA4-2	HPSGRRR	863.5	863.5	+3	-
mCA4-3	CHPSGKKKC	985.5	985.5	+3	-
mCA4-4	CHPSGRRRC	1069.5	1069.5	+3	-
mCA4-5	CIVIRFKFRC	1282.7	1281.7	+3	23%

The authors state that the scientific conclusions are unaffected. This correction was approved by the Academic Editor. The original publication has also been updated.
