# Supplementary material for: Bacterial-Specific Aggregation and Killing of Immunomodulatory Host Defense Peptides
Source: Pharmaceuticals (Basel). 2021 Aug 24;14(9):839. doi: 10.3390/ph14090839 (PMC8467575; doi:10.3390/ph14090839)
Supplement: Supplementary file 1 [file pharmaceuticals-14-00839-s001.zip › pharmaceuticals-1277883-supplementary.pdf]

Supporting Information

Bacterial Specific Aggregation and Killing of Immunomodulatory Host Defense Peptides

Nauman Nazeer[1], Juan Carlos Rodriguez-Lecompte[2], Marya Ahmed[1,3]

Department of Chemistry[1], Department of Pathology and Microbiology, Atlantic Veterinary College[2], Faculty of Sustainable Design Engineering[3]; University of Prince Edward Island, Charlottetown, Prince Edward Island, Canada, C1A 4P3

Corresponding email address: marahmed@upei.ca; jrodriguez@upei.ca

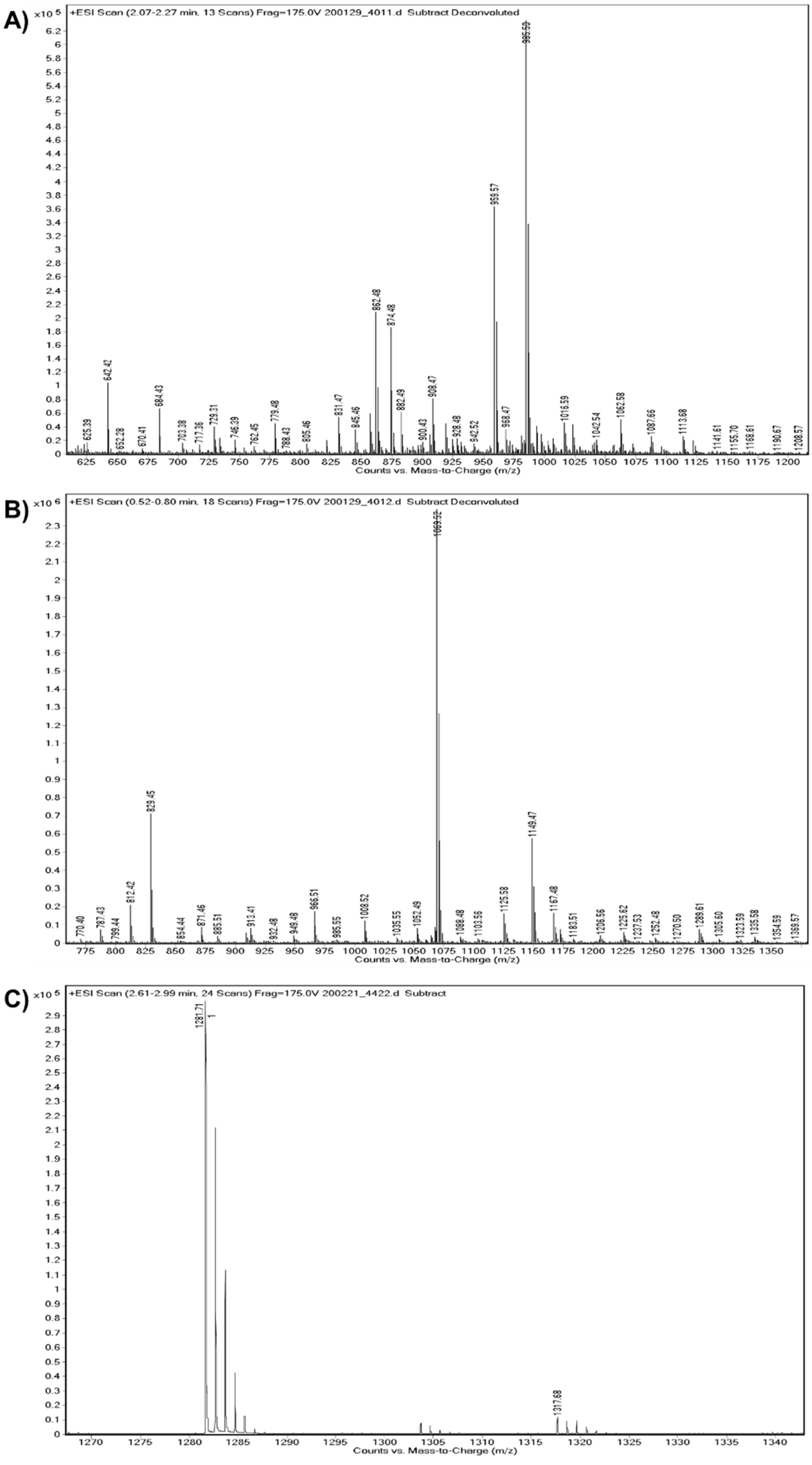
A)
+ESI Scan (2.07-2.27 min, 13 Scans) Frag=175.0V 200129_4011.d Subtract Deconvoluted
Counts vs. Mass-to-Charge (m/z)
B)
+ESI Scan (0.52-0.80 min, 18 Scans) Frag=175.0V 200129_4012.d Subtract Deconvoluted
Counts vs. Mass-to-Charge (m/z)
C)
+ESI Scan (2.61-2.99 min, 24 Scans) Frag=175.0V 200221_4422.d Subtract
Counts vs. Mass-to-Charge (m/z)

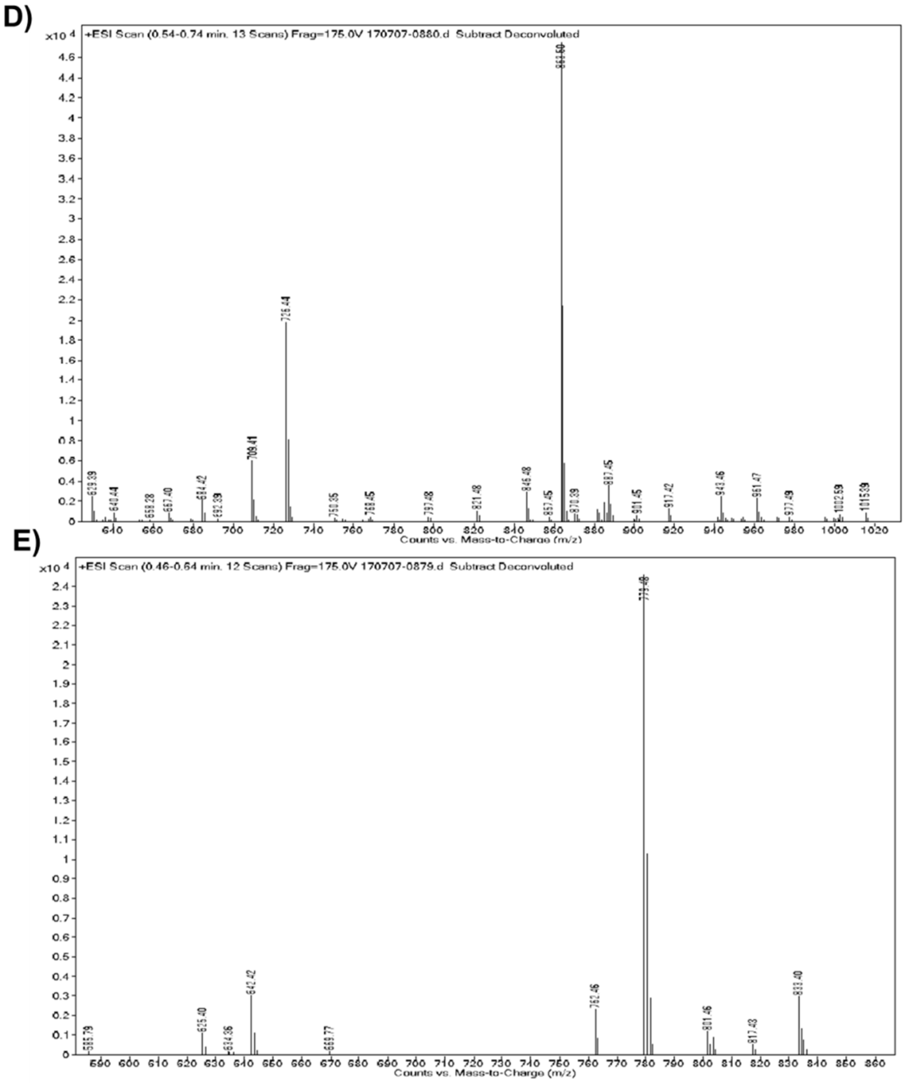

Figure S1: Mass spectrometry results for **(A)** mCA4-3; (**B)** mCA4-4; **(C)** mCA4-5; **(D)** mCA4-2; and **(E)** mCA4-1 peptides.

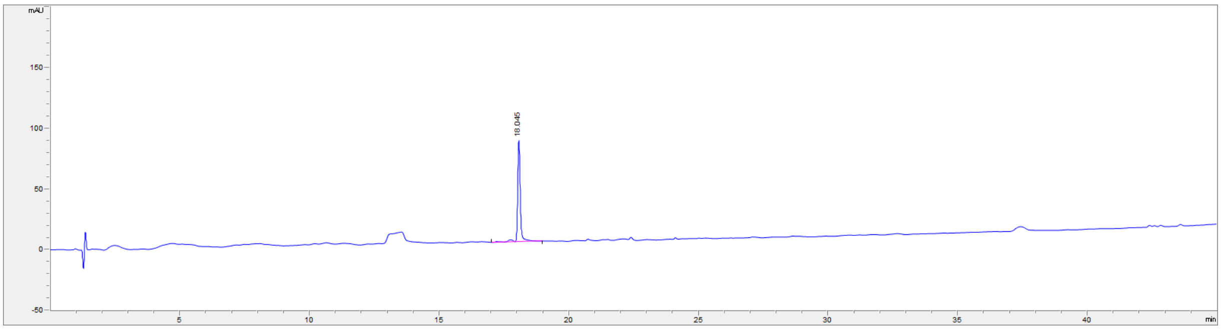

Figure S2: RP-HPLC chromatogram for mCA4-5 peptide.

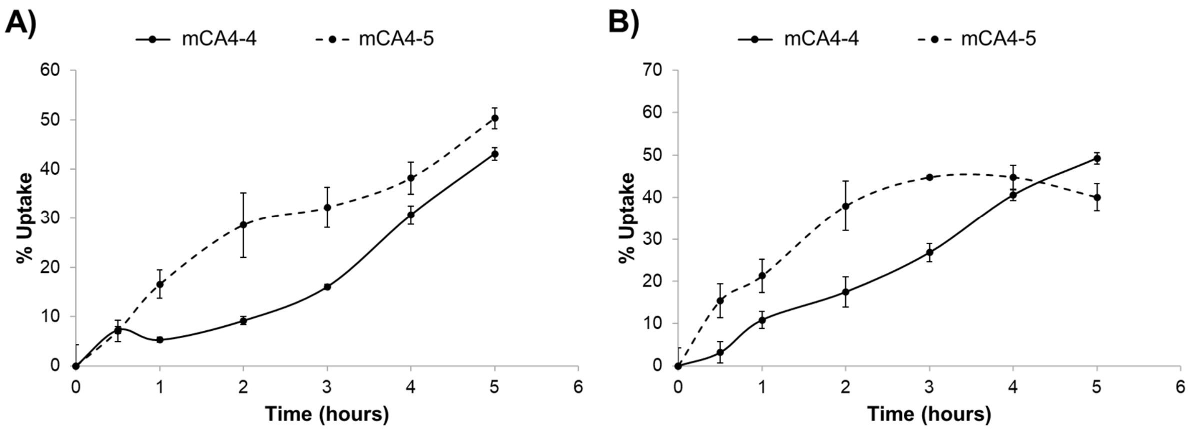

Figure S3: Uptake of selected AMPs in **(A)** *E. coli*; and **(B)** *B. subtilis*.

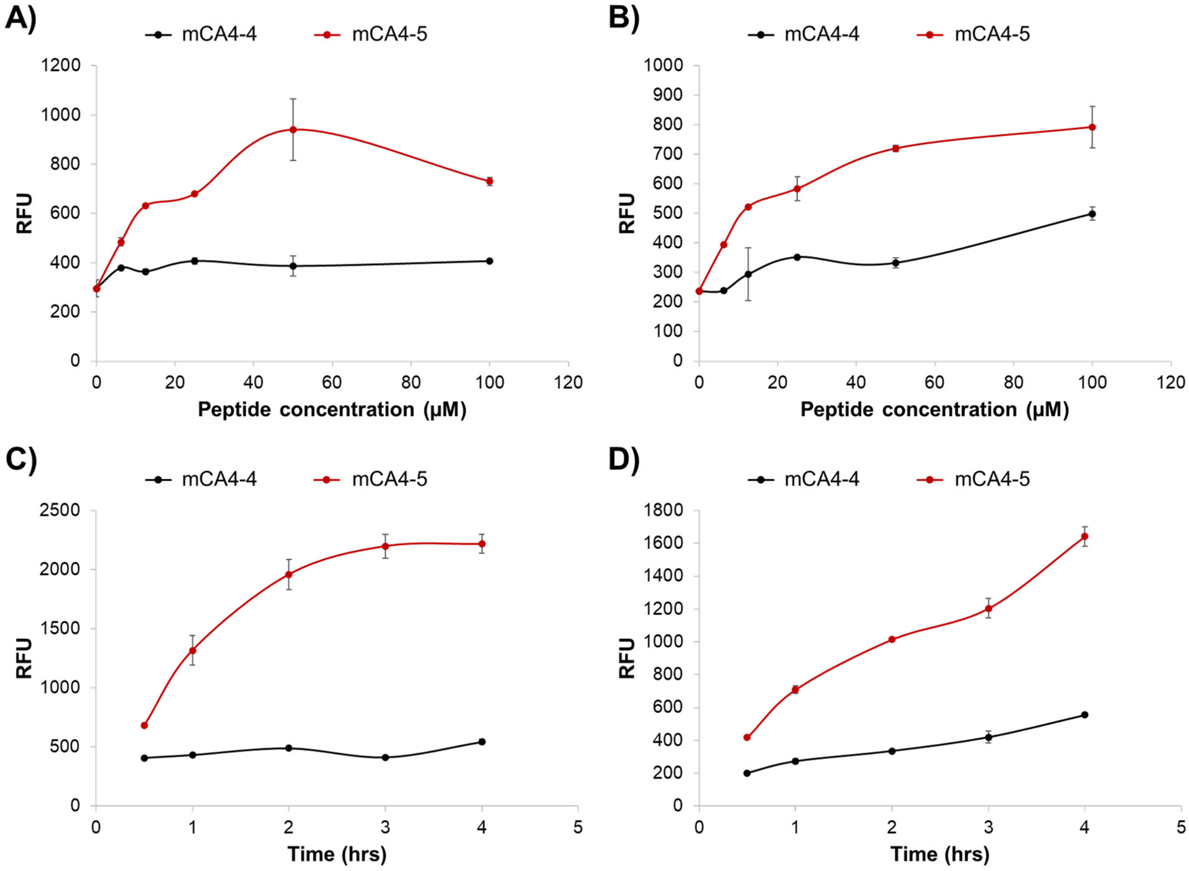

Figure S4: Membrane permeabilization efficacies AMPs in **(A & C)** *E. coli*; and **(B & D)** *B. subtilis*. A and B were done after a fixed time interval of 30 minutes. C and D were done using a fixed peptide concentration of 25μM.

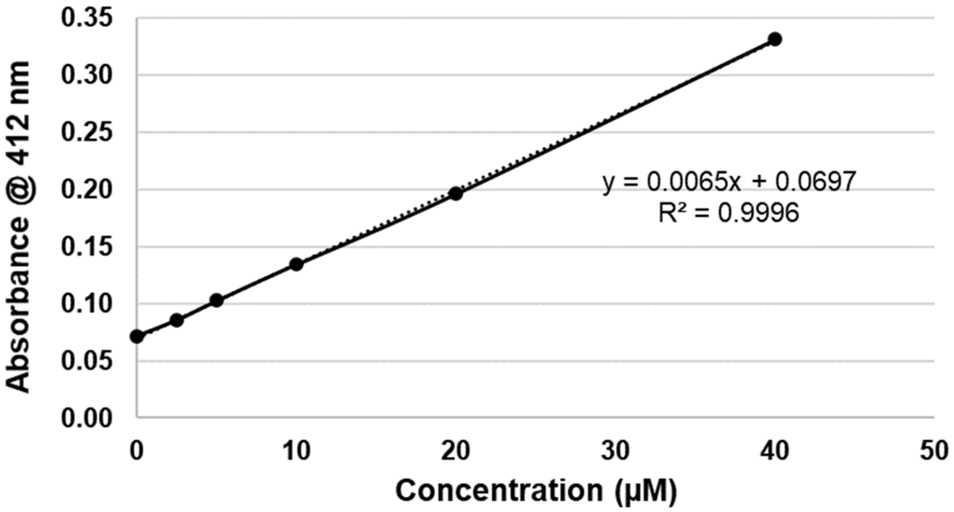

Sample	Mean Absorbance @ 420 nm
Blank	0.0718 ± 0.0004
mCA4-4 (40 μM)	0.087 ± 0.0002
mCA4-5 (40 μM)	0.0780 ± 0.001

Figure S5: Calibration of L-cysteine hydrochloride monohydrate by Elman's assay.

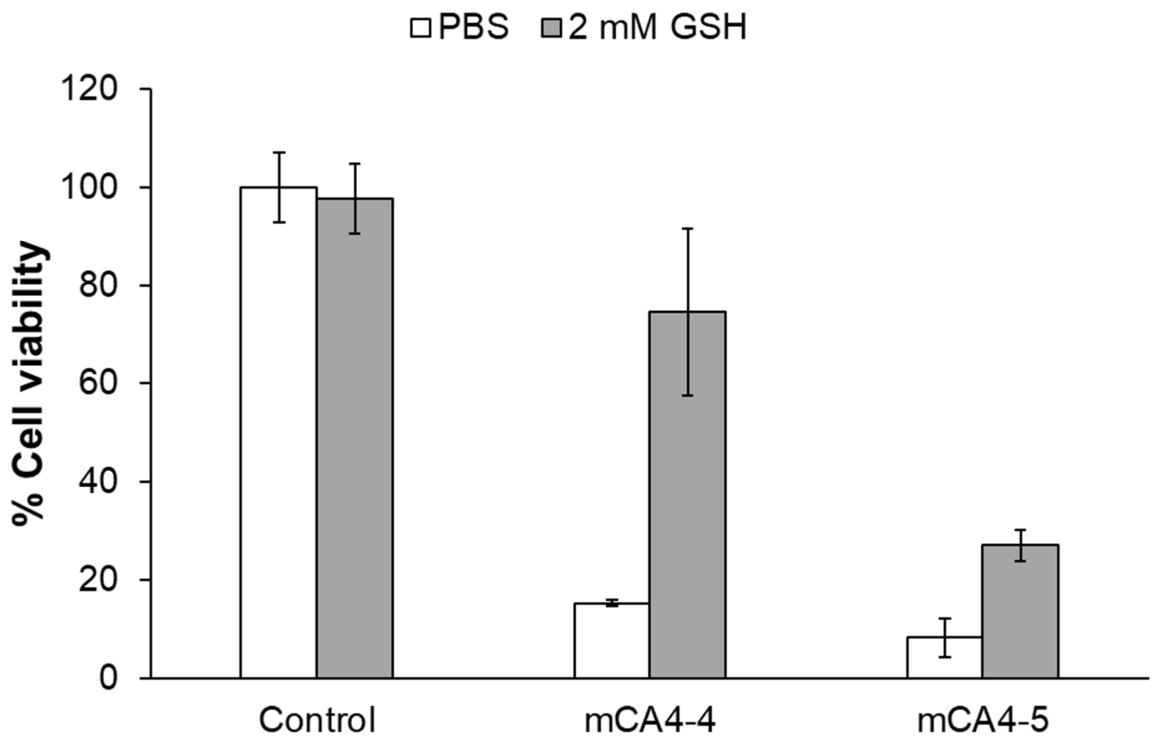

Figure S6: Effect of glutathione treatment on antimicrobial activity of peptides in *E. coli.* Peptide concentrations for mCA4-4 and mCA4-5 were 50 and 10 μM, respectively.

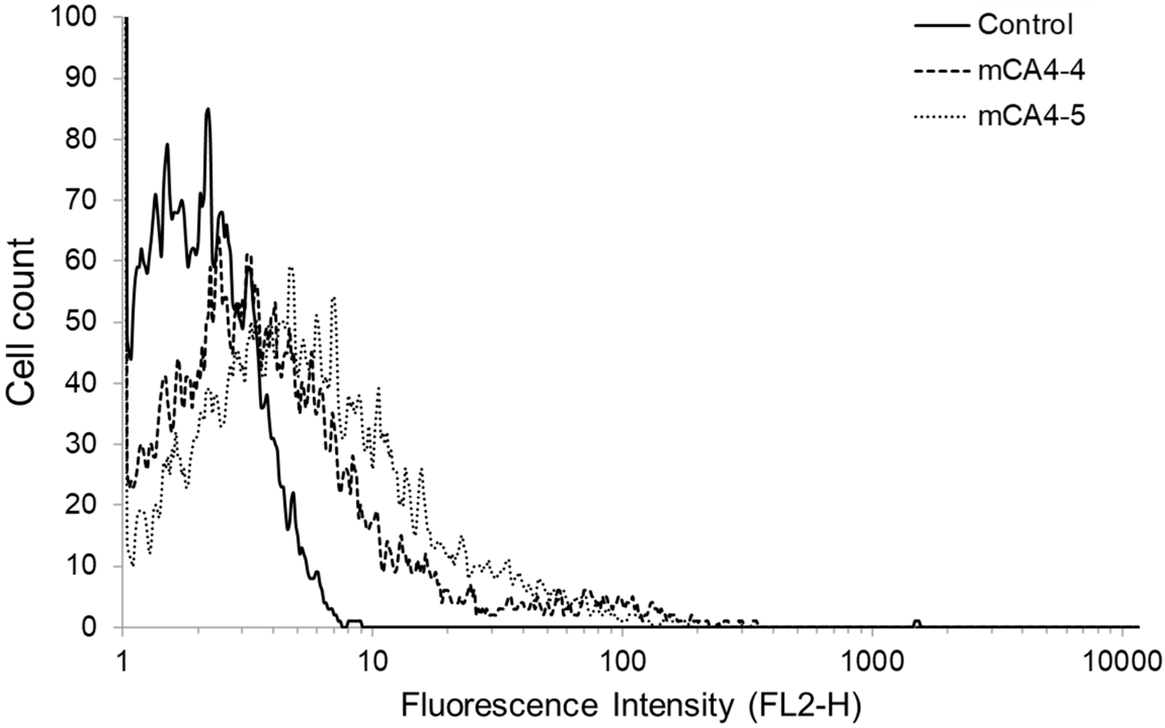

Sample	% Fluorescent cells
Control	–
mCA4-4–TAMRA	19.8%
mCA4-5–TAMRA	32.9%

Figure S7: Flow cytometry data indicating cellular uptake of peptides in Caco-2 cells.

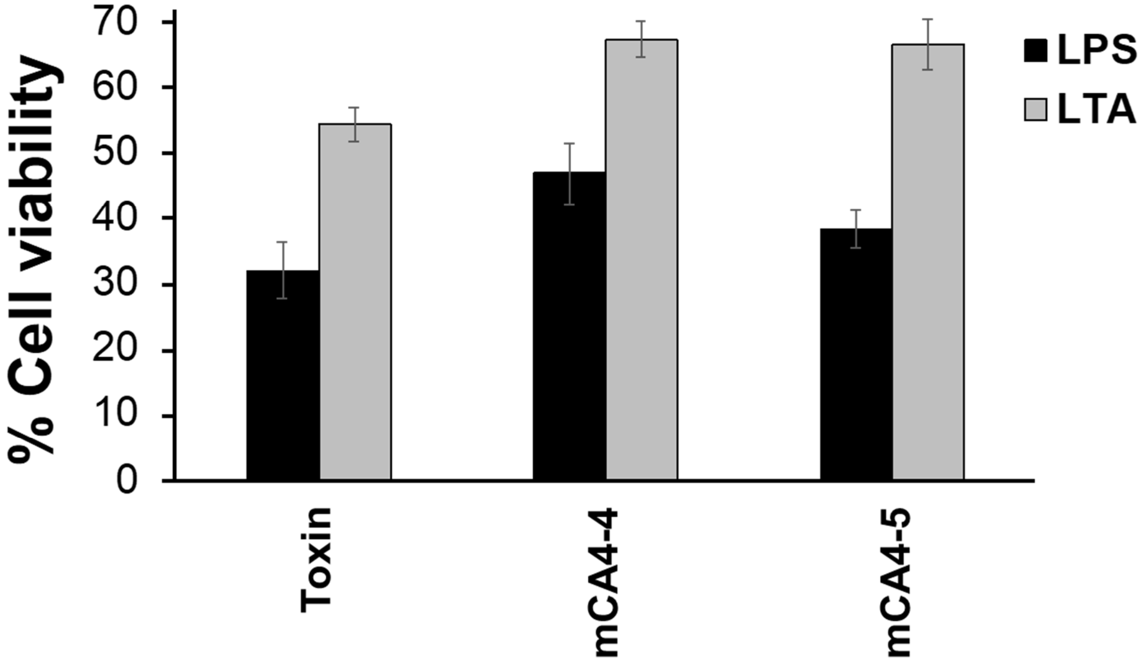

Figure S8: Toxin neutralization capability of peptides.

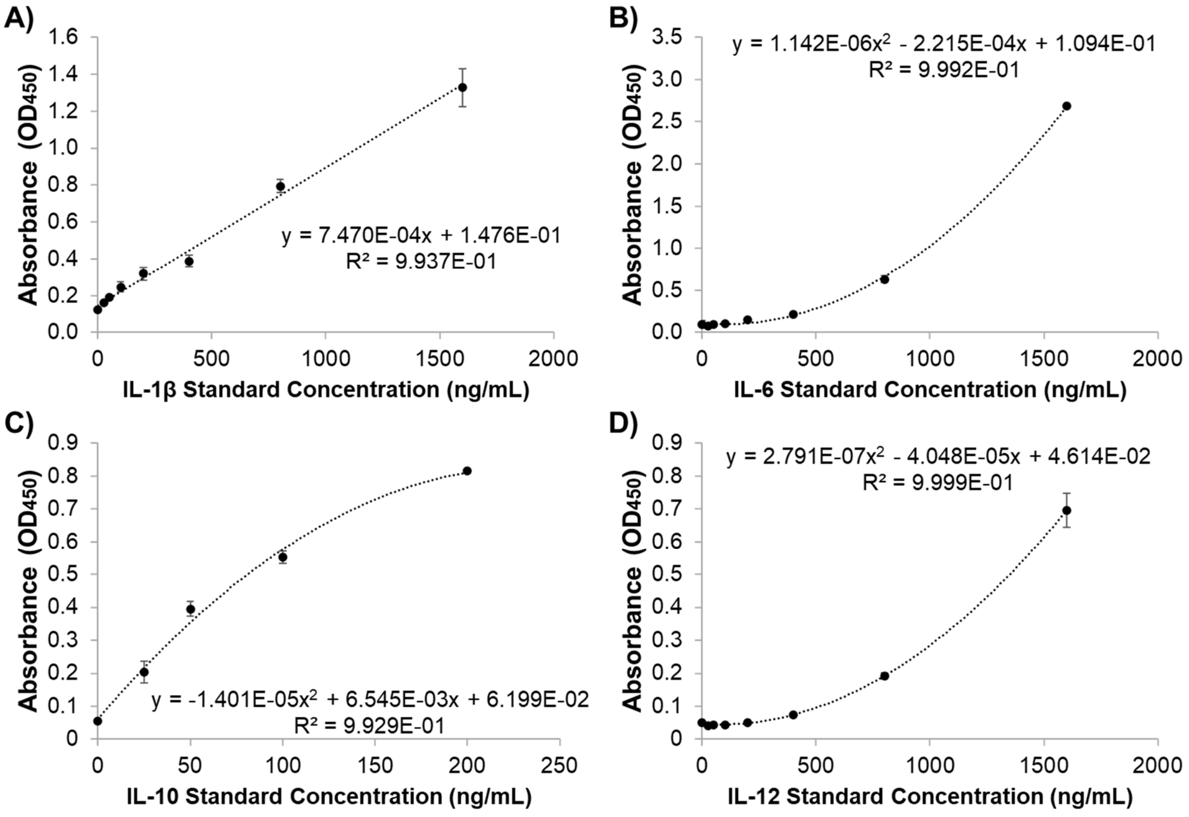

Figure S9: ELISA standard curves of **(A)** IL-1β; **(B)** IL-6; **(C)** IL-10; and **(D)** IL-12.
